# Medical Society in Wapello County

**Published:** 1855-07

**Authors:** 


### MEDICAL SOCIETY IN WAPELLO COUNTY.

We have received the proceedings of this Society, and take the occasion to express our regret that we are not able to give them a place in this number. We shall certainly find a place for them in our next. By organizing, the medical men of Wapello have placed themselves prominently forward before the medical profession, and it will give us pleasure always to give them the credit of being pioneers in county organization, and to aid them as far as possible in their praiseworthy efforts.

Well done, brethren of Wapello ; it will give us much pleasure at some future time, and that, too, not far hence, of taking each of you by the hand and bidding you God-speed in your praiseworthy efforts.

Will not other counties follow the example of Wapello? Will not the Empire county of Lee do so ? Is there not a necessity, a *stern* necessity for such an organization that we may be able to exchange opinions by discussing important topics connected with medical science, cultivate a spirit of friendship with those whose demanor is worthy of it, and expose charlatanism, *open* breaches of medical etiquette, examples of which are daily falling under our notice, and some of which we have recorded for future use. What say you, medical men of Lee? Will we organize? Our friends in Madison, West Point, Dover, Primrose, Charleston and elsewhere—will they unite with the medical men of Keokuk? We will see.

